# Associations between childhood trauma, cognitive function, and clinical symptoms in first-episode schizophrenia

**DOI:** 10.3389/fpsyt.2026.1911400

**Published:** 2026-09-09

**Authors:** Ru Wang, Xiang-Qin Qin, Xue-Qi Wang, Wen-Peng Hou, Chuan-Yue Wang, Xian-Bin Li

**Affiliations:** Beijing Key Laboratory of Mental Disorders, National Clinical Research Center for Mental Disorders and National Center for Mental Disorders, Beijing Anding Hospital, Capital Medical University, Beijing, China

**Keywords:** childhood trauma, clinical symptoms, cognitive function, physical neglect, schizophrenia

## Abstract

**Background:**

Childhood trauma may influence clinical presentation in first-episode schizophrenia (FES), but its specific associations with cognitive function and clinical symptoms remain unclear.

**Methods:**

Seventy-one FES participants and 62 healthy controls were assessed using the Childhood Trauma Questionnaire (CTQ-SF), MATRICS Consensus Cognitive Battery (MCCB), Positive and Negative Syndrome Scale (PANSS), and Calgary Depression Scale (CDSS). Group comparisons, Spearman correlations, hierarchical regression, and mediation analyses (PROCESS macro) were performed.

**Results:**

FES participants reported significantly higher childhood trauma (except physical abuse) and performed worse on all MCCB domains (all *p* < 0.00625). Within the clinical group, those with childhood trauma (71.8%) showed nominally lower MCCB total scores (*p* = 0.042) and a trend toward higher negative symptoms (*p* = 0.083). Exploratory analyses indicated that physical neglect was associated with attention/vigilance (r = -0.54) and overall cognition (*r* = -0.69); physical abuse with social cognition (*r* = -0.54); sexual abuse with general psychopathology (*r* = 0.56). No independent associations of trauma subtypes were found in regression analyses. Attention/vigilance was significantly associated with negative symptoms (β = -0.43, *p* = 0.002) but did not mediate the trauma-symptom relationship.

**Conclusion:**

FES participants report more childhood trauma and exhibit widespread cognitive impairment, with physical neglect most strongly linked to attentional deficits. Attention/vigilance was associated with negative symptoms, suggesting that trauma-informed cognitive rehabilitation may have potential value.

## Background

Schizophrenia is a chronic and severe mental disorder that leads to significant impairment in social functioning and reduced life expectancy, imposing a substantial burden on families and society (1–3). Cognitive impairment and negative symptoms are core features of schizophrenia and are closely associated with prognosis and functional outcomes in schizophrenia (4, 5). Therefore, investigating factors that influence cognitive function and clinical symptoms is of great importance.

Childhood trauma, including emotional abuse, physical abuse, sexual abuse, emotional neglect, and physical neglect, has been established as a significant environmental risk factor for schizophrenia (6, 7). Evidence suggests that participants with schizophrenia who have a history of childhood trauma may constitute a distinct subtype, characterized by more severe positive symptoms, higher suicide risk, and more pronounced cognitive impairments (8, 9). Among participants with first-episode schizophrenia, childhood trauma has been associated with more severe clinical symptoms and poorer cognitive function (10, 11).

A recent systematic review and meta-analysis supporting the “traumatogenic phenotype” hypothesis found that childhood trauma is significantly associated with more severe positive symptoms and cognitive deficits, particularly in executive function (12).Biological mechanism studies have suggested that childhood trauma may increase the risk of schizophrenia and influence its clinical presentation through effects on the hypothalamic-pituitary-adrenal axis function, neuroinflammatory responses, brain-derived neurotrophic factor levels, and brain structure development (13, 14). Animal model studies have also confirmed that early life stress can induce neurobiological alterations and behavioral abnormalities analogous to those observed in schizophrenia (15).

Despite growing attention to the relationship between childhood trauma and schizophrenia, studies specifically examining its impact on cognitive function in participants with first-episode schizophrenia remain relatively limited, and findings are inconsistent. Some studies have found that childhood trauma is associated with more severe cognitive impairment (10, 16), while others have not detected significant associations. Recent research suggests that the trauma-cognition relationship may not be simply linear (17), provided novel evidence for nonlinear associations between childhood trauma and cognitive function, which may account for discrepancies among existing studies. Furthermore, different trauma subtypes may exert differential effects on various domains of cognitive function and clinical symptoms (18, 19).

For instance, physical neglect is considered to have a particularly pronounced impact on cognitive function (13), while sexual abuse is more closely associated with general psychopathology (20). Network analysis approaches have also indicated that different types of trauma influence functional outcomes through distinct symptom pathways (21–26).However, systematic investigations examining the relationships between different trauma subtypes and multidimensional cognitive function and clinical symptoms specifically in participants with first-episode schizophrenia remain scarce.

Based on this, the present study aims to investigate the associations between childhood trauma, cognitive function and clinical symptoms in participants with first-episode schizophrenia, and to analyze the specific associations of different trauma subtypes. We hypothesized that: (1) participants would report higher levels of childhood trauma and demonstrate poorer cognitive function compared to healthy controls; (2) within the clinical group, those with childhood trauma would exhibit worse cognitive function and more severe clinical symptoms; (3) different dimensions of childhood trauma would be correlated to varying degrees with cognitive function and clinical symptoms; and (4) cognitive function might mediate the relationship between childhood trauma and clinical symptoms.

## Methods

### Participants

This study employed a case-control design. The clinical group was recruited from the outpatient and inpatient units of Beijing Anding Hospital, Capital Medical University, between July 2019 and June 2025. Inclusion criteria: (1) age 15–55 years, right-handed, normal hearing; (2) meeting DSM-IV criteria for schizophrenia, first-episode with illness duration <3 years; (3) education ≥9 years (junior high school or above); (4) providing written informed consent (or by legal guardian). Exclusion criteria: (1) history of alcohol or substance abuse; (2) neurological disorders or severe physical diseases; (3) pregnancy or breastfeeding; (4) electroconvulsive therapy or transcranial magnetic stimulation within the past 6 months; (5) any other Axis I disorder.

An illness duration of less than 3 years was used to identify individuals in the early phase of schizophrenia, consistent with previous duration-based definitions in the literature (27). Participants in the clinical group had a mean illness duration of 19.08 ± 13.44 months, indicating that the sample represented individuals in the early phase of schizophrenia.Healthy controls (n=62) were recruited via community advertisements. The same exclusion criteria applied, and they were screened with the Mini-International Neuropsychiatric Interview (MINI) to rule out any Axis I disorder.

### Instruments

Demographic questionnaire: self-designed to collect age, sex, years of education, marital status, ethnicity, etc.

Childhood Trauma Questionnaire-Short Form (CTQ-SF): The Chinese version (revised by Fu et al.) was used. It contains 28 items covering five subscales: emotional abuse (EA), physical abuse (PA), sexual abuse (SA), emotional neglect (EN), and physical neglect (PN), each ranging from 5 to 25. The clinical cutoffs for moderate-to-severe trauma were: EA ≥13, PA ≥10, SA ≥8, EN ≥15, PN ≥10.

MATRICS Consensus Cognitive Battery (MCCB): The Chinese version assesses seven cognitive domains: speed of processing, attention/vigilance, working memory, verbal learning, visual learning, reasoning and problem solving, and social cognition. A total score was also computed. Raw scores were converted to T-scores (mean=50, SD = 10).

Positive and Negative Syndrome Scale (PANSS): Used to evaluate clinical symptoms, including positive, negative, and general psychopathology subscales, and total score.

Calgary Depression Scale for Schizophrenia (CDSS): Assessed depressive symptoms in participants.

### Procedure

All participants were assessed by uniformly trained psychiatrists. Demographic data were collected first, followed by the subjects’ self-assessment using the CTQ-SF. For the clinical group, trained psychiatrists administered the PANSS and CDSS assessments. Additionally, all participants completed the MCCB cognitive assessment.

### Statistical analysis

Data were analyzed using SPSS 26.0 and the PROCESS macro. Group comparisons were performed using t-tests, Mann-Whitney U tests, or χ^2^ tests based on data distribution. Cognitive function was compared between groups using ANCOVA with years of education as a covariate, and partial η^2^ was calculated. Within the clinical group, participants were divided into trauma and non-trauma groups based on CTQ clinical cutoffs, and Mann-Whitney U tests were used to compare cognitive and clinical differences. Spearman correlation was used to analyze associations between variables. Hierarchical regression was performed to examine the independent associations of trauma, with collinearity diagnostics showing all VIFs < 2. Mediation analysis was conducted using PROCESS Model 4 with bootstrap method to calculate indirect effects. Bonferroni correction was applied for multiple comparisons, with significance levels set at p < 0.0083 for CTQ subscales, p < 0.00625 for MCCB domains, and p < 0.01 for clinical symptoms.

Before conducting the main analyses, data normality was examined using the Shapiro-Wilk test. Homogeneity of variances was assessed using Levene’s test. Multicollinearity was evaluated using variance inflation factors (VIFs), with all VIFs < 2, indicating no serious multicollinearity. Residual plots were inspected to verify model assumptions for regression and mediation analyses. For mediation, the PROCESS macro uses bootstrapping, which is robust to non-normality for indirect effect estimation.

No formal *a priori* sample size calculation was performed because this study was based on an available clinical sample with an exploratory design. The relatively small sample size may have limited statistical power, particularly for subgroup comparisons, correlation analyses, and mediation analyses.

## Results

### Demographic characteristics

A total of 71 participants with first-episode schizophrenia and 62 healthy controls were included in this study. There were no significant differences between the two groups in age, sex, marital status, or ethnicity (p > 0.05), indicating good comparability on these basic characteristics. However, the control group had significantly higher years of education than the clinical group (p = 0.008), which was therefore controlled as a covariate in subsequent analyses of cognitive function. Detailed demographic characteristics are presented in **Table 1**.

**Table 1:** Demographic characteristics.

Variable	Clinical group (n=71)	Control group (n=62)	Statistic	p-value
Age (years)	25.52 ± 6.68	25.21 ± 3.82	t = 0.335	0.738
Illness duration (months)	19.08 ± 13.44	—	—	—
Education (years)	12.00 (11.00-16.00)	15.00 (12.00-17.00)	U = 1615.500	0.008
Sex (male, %)	33 (46.5%)	35 (56.5%)	χ^2^ = 1.320	0.251
Marital status (no spouse, %)	57 (80.3%)	51 (82.3%)	χ^2^ = 0.085	0.771
Ethnicity (Han, %)	69 (97.2%)	60 (96.8%)	χ^2^ = 0.020	0.991

Data are presented as mean ± standard deviation or median (interquartile range); categorical variables are presented as frequency (%). *p*-values were derived from independent t-tests, Mann-Whitney U tests, or χ^2^ tests.

### Comparison of childhood trauma reports

Mann-Whitney *U* tests (**Table 2**) showed that participants scored significantly higher than controls on the CTQ total score and most subscales. After Bonferroni correction (p < 0.0083), the clinical group had significantly higher scores on CTQ total, emotional abuse, sexual abuse, emotional neglect, and physical neglect (p < 0.001 to 0.001), while physical abuse did not differ between groups (p = 0.232). This suggests that participants with first-episode schizophrenia reported more childhood trauma, particularly in the domains of emotional abuse, sexual abuse, emotional neglect, and physical neglect.

**Table 2:** Comparison of childhood trauma reports (CTQ).

Variable	Clinical group (n=71)	Control group (n=62)	U value	p-value
CTQ Total Score	45.00 (36.50-52.00)	34.00 (30.00-39.00)	994.5	<0.001
EA	8.00 (6.00-10.00)	6.00 (5.00-7.00)	1328.0	<0.001
PA	5.00 (5.00-7.00)	5.00 (5.00-6.00)	1969.5	0.232
SA	5.00 (5.00-7.25)	5.00 (5.00-5.00)	1546.5	0.001
EN	13.00 (9.00-19.00)	9.00 (6.75-13.00)	1180.0	<0.001
PN	10.00 (8.00-13.00)	8.00 (6.00-9.25)	1228.5	<0.001

Data are presented as median (interquartile range). *p*-values were derived from Mann-Whitney U tests. Bonferroni correction was applied with a significance level of *p* < 0.0083. CTQ subscale abbreviations: EA, Emotional Abuse, PA, Physical Abuse, SA, Sexual Abuse, EN, Emotional Neglect, PN, Physical Neglect.

### Comparison of cognitive function (MCCB)

ANCOVA with years of education as a covariate (**Table 3**) indicated that, after Bonferroni correction (p < 0.00625), the clinical group performed significantly worse than the control group on all MCCB cognitive domains and the total score (p < 0.001 to 0.005). Effect sizes (partial η^2^) ranged from 0.067 to 0.401. The largest group differences were observed in speed of processing and attention/vigilance (partial η^2^ > 0.35). These results indicate widespread cognitive impairment in participants with first-episode schizophrenia.

**Table 3:** Comparison of cognitive function (MCCB).

MCCB domain	Clinical group (n=71)	Control group (n=62)	F value	p-value	partial η^2^
Speed of Processing	33.86 ± 0.971	44.37 ± 0.912	60.318	<0.001	0.350
Attention/Vigilance	30.62 ± 1.311	45.88 ± 1.183	72.279	<0.001	0.401
Working Memory	38.83 ± 1.126	46.29 ± 1.067	22.370	<0.001	0.165
Verbal Learning	38.83 ± 1.181	47.06 ± 1.129	24.632	<0.001	0.180
Visual Learning	40.14 ± 1.604	46.49 ± 1.507	8.085	0.005	0.067
Reasoning & Problem Solving	34.95 ± 1.410	42.90 ± 1.336	16.243	<0.001	0.126
Social Cognition	32.31 ± 1.502	38.77 ± 1.435	9.385	0.003	0.079
MCCB Total Score	36.14 ± 0.793	44.53 ± 0.712	60.322	<0.001	0.372

Data are presented as estimated marginal means ± standard error from ANCOVA with years of education as a covariate. *p*-values were Bonferroni-corrected with a significance level of *p* < 0.00625. Partial η^2^ indicates effect size.

### Comparison of trauma vs. non-trauma subgroups within the clinical group

Based on CTQ clinical cutoffs (emotional abuse ≥13, physical abuse ≥10, sexual abuse ≥8, emotional neglect ≥15, physical neglect ≥10), participants were divided into a trauma group (n=51, 71.8%) and a non-trauma group (n=20, 28.2%). Mann-Whitney *U* tests (**Table 4**) showed:

**Table 4:** Comparison of cognitive function and clinical symptoms between trauma and non-trauma subgroups within the clinical group.

Variable	Trauma group (n=51)	Non-trauma group (n=20)	U value	p-value
Cognitive Function				
Speed of Processing	31.00 (26.00-37.00)	35.00 (30.00-40.00)	210.0	0.110
Attention/Vigilance	28.00 (23.00-38.00)	32.00 (24.00-37.00)	245.0	0.711
Working Memory	37.00 (32.00-44.00)	42.00 (37.00-47.00)	230.0	0.128
Verbal Learning	37.00 (30.00-44.00)	42.50 (36.25-46.75)	233.5	0.145
Visual Learning	40.50 (25.75-49.25)	46.00 (33.25-50.00)	267.5	0.489
Reasoning & Problem Solving	30.00 (26.00-40.00)	35.00 (26.25-46.25)	269.5	0.431
Social Cognition	28.50 (21.75-37.25)	40.00 (20.75-45.00)	241.5	0.236
MCCB Total Score	34.00 (29.00-39.00)	38.00 (34.00-41.25)	144.0	*0.042*
Clinical Symptoms				
Positive Symptoms	23.00 (18.00-27.00)	24.00 (21.00-27.75)	472.5	0.631
Negative Symptoms	23.00 (15.00-27.00)	16.50 (10.50-25.50)	374.5	0.083
General Psychopathology	41.00 (37.00-46.00)	41.00 (38.00-43.00)	455.5	0.485
PANSS Total Score	84.00(76.00-97.00)	80.50(73.00-90.75)	404.0	0.175
CDSS Total Score	1.00 (0.00-3.00)	0.50 (0.00-2.00)	409.0	0.225

Data are presented as median (interquartile range). *p*-values were derived from Mann-Whitney U tests. *p < 0.05 (nominal significance), but did not remain significant after Bonferroni correction (cognitive domains: *p* < 0.00625; clinical symptoms: *p* < 0.01).

**Figure 1:**
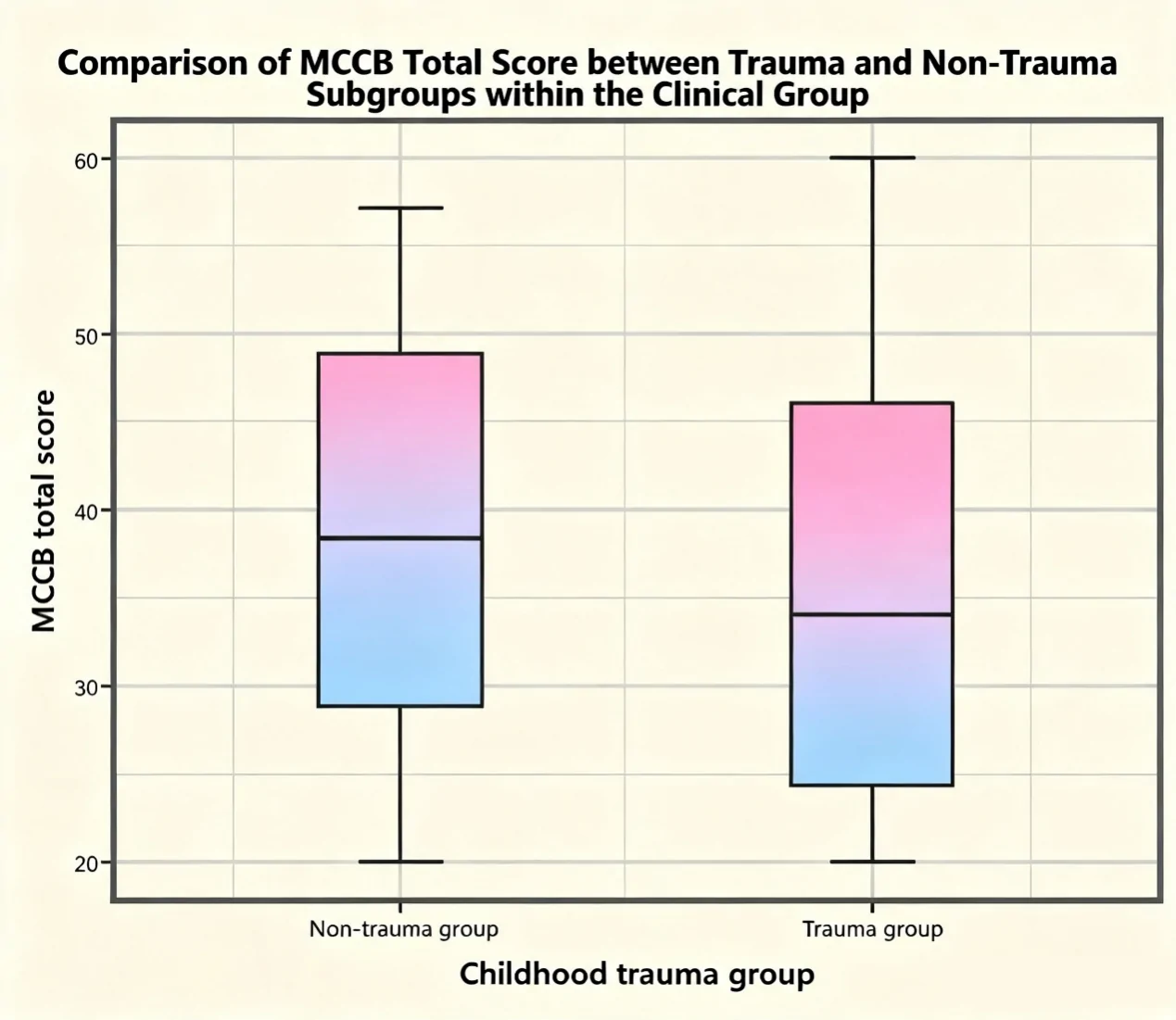
Exploratory comparison of MCCB total scores between trauma and non-trauma subgroups.

Cognitive Function: The trauma group had a lower MCCB total score than the non-trauma group (*p* = 0.042, **Figure 1**), but this did not survive Bonferroni correction (*p* < 0.00625). No significant differences were found in other cognitive domains (*p* > 0.05).

**Figure 2:**
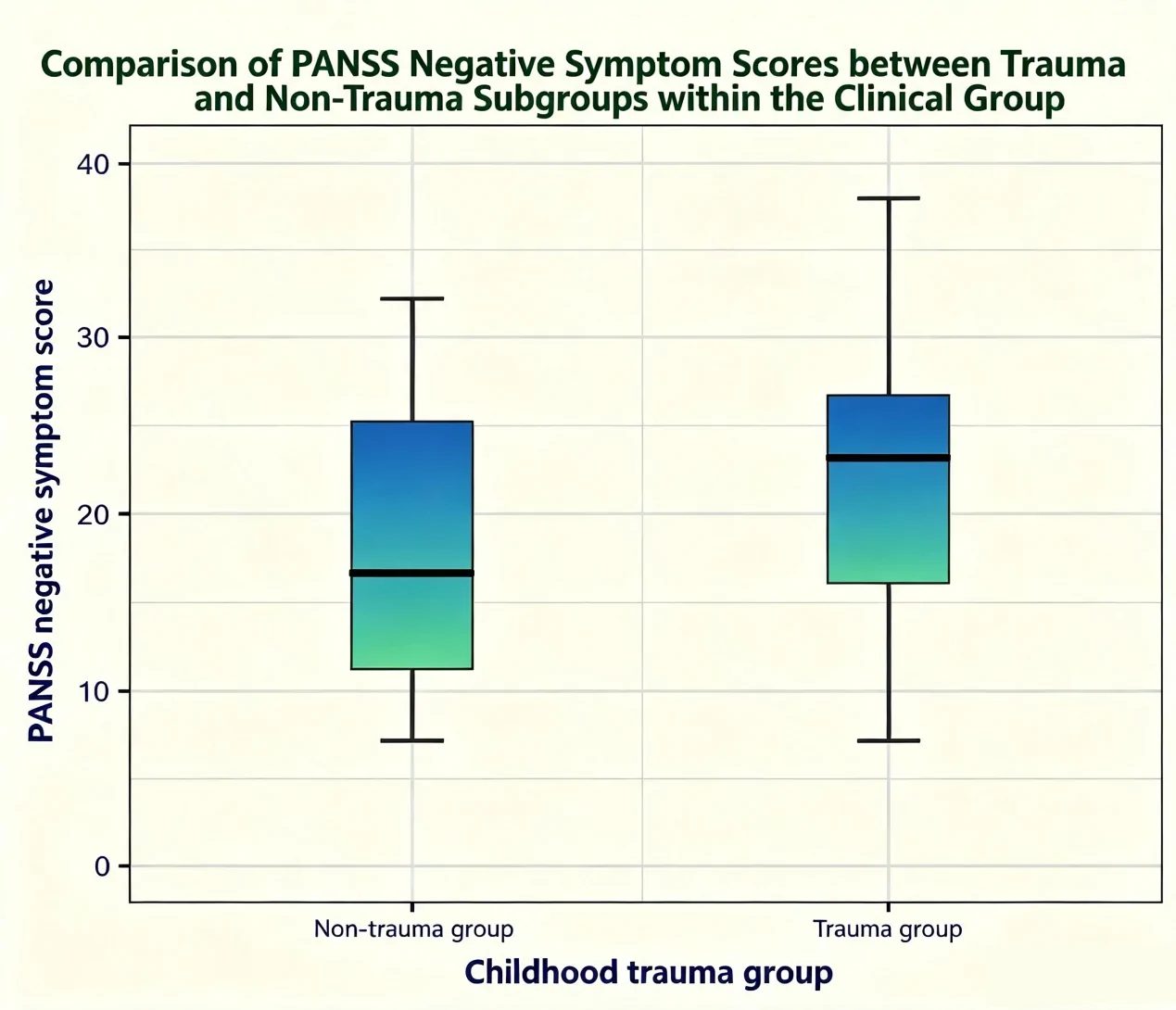
Exploratory comparison of PANSS negative symptom scores between trauma and non-trauma subgroups.

Clinical Symptoms: The trauma group showed higher negative symptoms compared to the non-trauma group, approaching significance (*p* = 0.083, **Figure 2**). No significant differences were found in positive symptoms, general psychopathology, PANSS total score, or CDSS total score (*p* > 0.05).

### Correlation analysis of childhood trauma with cognitive function and clinical symptoms

Spearman correlation analyses (**Tables 5** and **6**) indicated the following:

**Table 5:** Correlation analysis between childhood trauma and cognitive function (Spearman’s rho).

Variable	EA	PA	SA	EN	PN	CTQ total
Speed of Processing	-.439	-.155	-.250	.072	-.412	-.340
Attention/Vigilance	-.206	-.133	-.068	.309	-.541*	-.102
Working Memory	.428	.240	-.206	.525*	.077	.435
Verbal Learning	-.035	-.311	-.412	-.145	-.371	-.190
Visual Learning	-.033	-.412	-.246	-.105	-.335	-.121
Reasoning & Problem Solving	-.423	.152	-.185	-.054	-.226	-.369
Social Cognition	-.273	-.539*	.329	-.058	-.225	-.210
MCCB Total Score	-.352	-.277	-.356	.114	-.692**	-.337

Values are Spearman’s correlation coefficients. *p < 0.05, **p < 0.01 (uncorrected). These analyses were not corrected for multiple comparisons; findings are exploratory and require validation.

**Table 6:** Correlation analysis between childhood trauma and clinical symptoms (Spearman’s rho).

Variable	EA	PA	SA	EN	PN	CTQ total
PANSS Positive	.350	.310	.282	.156	-.043	.259
PANSS Negative	-.039	.028	.080	-.102	-.042	-.075
PANSS General Psychopathology	.090	-.085	.563**	-.178	-.069	.045
PANSS Total	.049	.149	.431	-.273	-.090	-.024
CDSS Total	.132	.182	.099	.079	-.017	.025

Values are Spearman’s correlation coefficients. *p < 0.05, **p < 0.01 (uncorrected). These analyses were not corrected for multiple comparisons; findings are exploratory and require validation.

**Figure 3:**
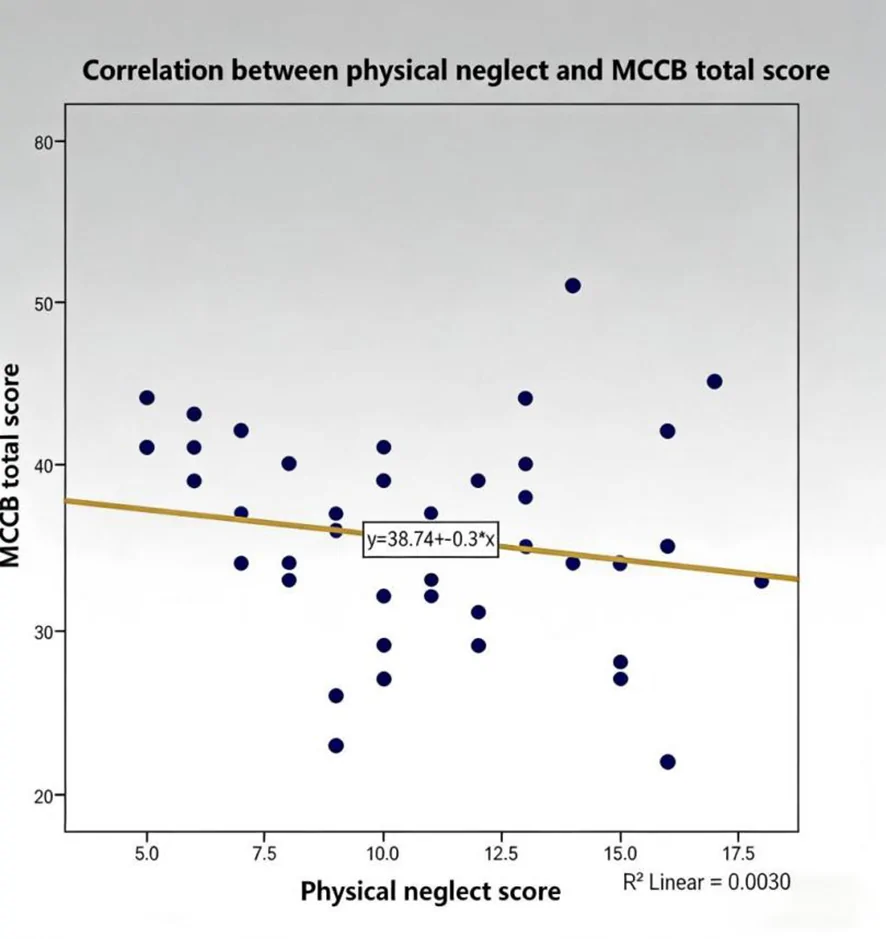
Correlation between physical neglect and MCCB total score.

Cognitive Function: Exploratory analyses indicated that physical neglect was associated with attention/vigilance (*r* = -0.54, *p* < 0.05) and MCCB total score (*r* = -0.69, *p* < 0.01, **Figure 3**); physical abuse was associated with social cognition (*r* = -0.54, *p* < 0.05); and emotional neglect was associated with working memory (*r* = 0.53, *p* < 0.05). The CTQ total score was also significantly negatively correlated with attention/vigilance and MCCB total score (*p* < 0.05).

Clinical Symptoms: Sexual abuse was significantly positively correlated with PANSS general psychopathology (*r* = 0.56, *p* < 0.01). No other trauma dimensions showed significant correlations with clinical symptoms.

### Regression analysis of childhood trauma and cognitive function and clinical symptoms

Hierarchical regression analyses, controlling for demographic variables (**Table 7**), showed that none of the CTQ dimensions significantly predicted MCCB total score, PANSS negative symptoms, PANSS total score, or CDSS total score (all ΔR^2^ *p* > 0.05, all β *p* > 0.05).

**Table 7:** Hierarchical multiple regression analysis of childhood trauma and cognitive function and clinical symptoms (standardized coefficients β).

Predictor	MCCB total score	PANSS negative symptoms	PANSS total score	CDSS total score
Model 1				
Sex	-0.101	-0.065	0.187	-0.023
Education	0.305	-0.205	-0.280	0.257
Ethnicity	0.039	0.055	-0.116	-0.122
Age at Onset	0.329	0.009	-0.006	0.106
Model 2				
EA	0.111	0.004	-0.089	0.213
PA	-0.150	0.049	0.061	0.040
SA	-0.203	-0.141	-0.089	-0.051
EN	0.060	-0.265	-0.134	0.072
PN	-0.040	0.345	0.234	0.045
R^2^ (Model 1)	0.251	0.070	0.119	0.097
ΔR^2^ (Model 2)	0.078	0.099	0.045	0.069
Total R^2^	0.329	0.169	0.165	0.166

Values are standardized regression coefficients (β). All *p* > 0.05. Variance inflation factors (VIF) for all predictors were < 2, indicating no serious multicollinearity. Model 1 controlled for demographic variables (sex, education, ethnicity, age at onset); Model 2 added CTQ subscales. ΔR^2^ represents the increment in variance explained by Model 2 compared to Model 1.

### Mediation analysis of attention/vigilance in the relationship between physical neglect and negative symptoms

A mediation analysis was conducted using Hayes’ PROCESS macro (Model 4) with 5000 bootstrap samples to test whether attention/vigilance mediated the relationship between physical neglect and negative symptoms, controlling for age at onset and education. Missing data primarily arose from incomplete responses on individual subtests of the MCCB attention/vigilance domain. Complete-case analysis (listwise deletion, the default approach in PROCESS) was used, including only the 50 participants with full data on all analysis variables. The overall regression model explaining negative symptoms was statistically significant (R^2^ = 0.20, F(4,45) = 2.81, p = 0.036). The Durbin-Watson statistic was 1.92, indicating independence of errors, and residual plots did not reveal significant departures from normality or homoscedasticity.

The results (**Table 8**) showed that physical neglect did not significantly predict attention/vigilance (β = 0.07, *p* = 0.635). However, attention/vigilance was significantly negatively associated with negative symptoms (β = -0.43, *p* = 0.0016). The direct effect of physical neglect on negative symptoms approached significance (β = 0.24, *p* = 0.078), while the total effect was not significant (β = 0.21, *p* = 0.161). The indirect effect was -0.031 with a 95% bootstrap confidence interval of [-0.16, 0.09], which includes zero, indicating that attention/vigilance did not mediate the relationship between physical neglect and negative symptoms.

**Table 8:** Mediation analysis results (standardized coefficients).

Path	Effect	Boot SE	Boot LLCI	Boot ULCI
Total Effect	0.21	0.15	-0.09	0.50
Direct Effect	0.24	0.13	-0.03	0.50
Indirect Effect	-0.031	0.061	-0.16	0.09

Values are standardized effects. BootLLCI and BootULCI are the lower and upper limits of the 95% bootstrap confidence intervals based on 5000 resamples. Due to missing data, the effective sample size for mediation analysis was 50.

## Discussion

This study systematically examined the associations between childhood trauma and cognitive function and clinical symptoms in participants with first-episode schizophrenia. The main findings include: (1) The clinical group reported significantly higher levels of childhood trauma than healthy controls, particularly in emotional abuse, sexual abuse, emotional neglect, and physical neglect; (2) The clinical group exhibited widespread cognitive impairment, with the most severe deficits in speed of processing and attention/vigilance; (3) Within the clinical group, the trauma subgroup showed a trend toward lower overall cognitive function and higher negative symptoms, although these did not reach statistical significance after multiple comparison correction; (4) Correlation analyses indicated that physical neglect was associated with attention/vigilance and overall cognitive function, physical abuse was negatively correlated with social cognition, and sexual abuse was positively correlated with general psychopathology; (5) Regression analyses did not find independent predictive effects of any childhood trauma dimension on cognition or symptoms; (6) Mediation analysis showed that attention/vigilance significantly negatively predicted negative symptoms but did not mediate the relationship between physical neglect and negative symptoms.

### Childhood trauma and cognitive function: participant-control comparisons

Consistent with previous research, participants with first-episode schizophrenia reported significantly higher levels of childhood trauma than healthy controls (28, 29). As an important environmental risk factor for schizophrenia, childhood trauma may increase disease susceptibility through effects on neurodevelopment and stress response systems (30–32). Simultaneously, the clinical group performed significantly worse than controls on all MCCB cognitive domains, with moderate to large effect sizes, consistent with the view that cognitive impairment is a core feature of schizophrenia. Notably, the effect sizes for speed of processing and attention/vigilance were largest (partial η^2^ > 0.35), suggesting that these basic cognitive processes are severely impaired in the early stages of illness, consistent with previous studies (10, 16, 33).

### Differences between trauma and non-trauma subgroups within the clinical group

Within the clinical group, the trauma subgroup (71.8%), defined according to clinical cutoffs, had a lower MCCB total score than the non-trauma subgroup (nominal p = 0.042) and showed a trend toward higher negative symptoms (p = 0.083); however, these differences did not reach statistical significance after Bonferroni correction. Previous research has also suggested a potential positive association between childhood trauma and negative symptoms (34), a trend partially corroborated by the present study. The lack of significance may be related to several factors: First, the non-trauma subgroup had only 20 participants, resulting in insufficient statistical power and increased risk of type II errors. Second, although Bonferroni correction controls for type I errors, it also increases the risk of type II errors. Third, dichotomization may lose continuous information on trauma severity, thereby reducing statistical power. Future studies with larger samples are needed to validate these trends.

### Specific associations between childhood trauma subtypes and cognitive function and clinical symptoms

Correlation analyses indicated specific associations between different trauma subtypes and cognitive and clinical domains, providing clues for understanding the heterogeneous associations of trauma subtypes with cognitive and clinical outcomes.

The strong negative correlation between physical neglect and attention/vigilance and overall cognition was one of the notable exploratory associations observed in this study. Physical neglect refers to caregivers’ failure to meet an individual’s basic physical needs, which may have widespread and lasting effects on the developing brain. Previous research suggests that physical neglect is associated with reduced hippocampal and prefrontal cortex volumes (35, 36), brain regions critical for attention and cognitive control. Wang et al. (13) reported that physical neglect leads to cognitive deficits through effects on orbitofrontal cortex H-shaped sulci volume. Furthermore, childhood trauma can dysregulate HPA axis function, leading to abnormal cortisol levels and subsequent impairment of hippocampus-dependent cognitive function (37). Animal models have also confirmed that early life stress can induce neurobiological changes analogous to those observed in schizophrenia (38).

The significant negative correlation between physical abuse and social cognition is consistent with studies by Crawford et al. (39). Social cognition involves complex processes such as understanding others’ intentions and emotion management, with neural underpinnings including the medial prefrontal cortex and superior temporal gyrus. Duque-Alarcón et al. (40) found that childhood abuse is associated with hyperconnectivity in the medial prefrontal cortex, which may underlie social cognitive impairment. Ferrer et al. (41) further suggested that the interaction between FKBP5 gene polymorphisms and physical abuse affects social cognition, highlighting the importance of gene-environment interactions.

The positive correlation between sexual abuse and general psychopathology aligns with previous research (20); sexual abuse is often associated with more complex clinical symptoms, including depression, anxiety, and post-traumatic stress symptoms, possibly related to emotion dysregulation and amygdala hyperactivation (42). Notably, although the prevalence of sexual abuse was low in this sample (median 5), its impact may be particularly severe when it occurs.

The positive correlation between emotional neglect and working memory was opposite to the expected direction and should be interpreted cautiously, possibly reflecting statistical chance or confounding factors. Emotional neglect often co-occurs with other types of trauma and may manifest as a compensatory effect on working memory, or may be related to sample characteristics; this finding requires validation in independent samples.

### Regression analyses: why no independent predictive effects were found

Hierarchical regression analyses showed that after controlling for demographic variables, none of the CTQ dimensions significantly predicted cognition or symptoms. This inconsistency with the significant correlations observed in bivariate analyses may be due to the following reasons: (1) Multicollinearity: Moderate correlations exist among CTQ dimensions (e.g., between emotional neglect and physical neglect), which compete for variance in regression models, attenuating their unique contributions. (2) Sample size limitations: With 71 participants and 8 predictors (4 control variables + 5 trauma dimensions), statistical power was limited for detecting small to medium effect sizes. (3) Effects of control variables: Age at onset and years of education are themselves closely related to cognition and symptoms and may have explained variance that would otherwise be attributed to trauma. (4) Nonlinear relationships: Denaroso et al. (17) recently proposed that trauma-cognition associations may be nonlinear, and linear regression models may not adequately capture these relationships.

It should be noted that the present study only examined linear relationships between childhood trauma and cognitive function. Denaroso et al. (17) reported nonlinear associations, such that cumulative trauma, emotional neglect, and emotional abuse showed nonlinear relationships with cognition. Our regression models were based on linear assumptions and may not fully capture such complex relationships. Future studies should employ segmented regression or quadratic term approaches to explore potential threshold effects—that is, at what level of trauma severity the impact on cognition changes.

### Association between attention/vigilance and negative symptoms

Mediation analysis indicated that attention/vigilance significantly negatively predicted negative symptoms (β = -0.43, p = 0.002), a notable finding of this exploratory study. Attention/vigilance, as a fundamental component of cognitive function, has previously been associated with negative symptoms (such as apathy and social withdrawal) (43), suggesting that cognitive training may improve negative symptoms. However, attention/vigilance did not mediate the relationship between physical neglect and negative symptoms, primarily because the path from physical neglect to attention/vigilance was not significant. This path was significant in correlation analyses (r = -0.54) but lost significance in the mediation model due to the inclusion of covariates (age at onset, education) and reduced sample size (50 cases). This suggests that the effect of physical neglect on attention/vigilance may be explained by other variables or may exhibit threshold effects (only affecting attention when reaching a certain severity). Furthermore, the direct effect of physical neglect on negative symptoms approached significance (p = 0.078), suggesting the presence of other unmeasured mediators, such as emotion regulation abilities, social support, or biological markers (e.g., BDNF, inflammatory factors) (13, 14, 44). Because the bootstrap 95% confidence interval of the indirect effect included zero, the mediation hypothesis was not supported.

### Clinical implications

The findings of this study have several implications for clinical practice. First, given the high prevalence of childhood trauma in participants with first-episode schizophrenia, routine clinical assessment should include screening for trauma history, particularly physical neglect, emotional neglect, and sexual abuse. Second, the strong association between physical neglect and attention/vigilance and overall cognitive impairment suggests that participants with a history of physical neglect may particularly benefit from attention assessment and cognitive rehabilitation. Third, the association between sexual abuse and general psychopathology indicates that participants with a history of sexual abuse require close monitoring for symptoms such as depression and anxiety, as well as trauma-informed care. Fourth, the observed association between attention/vigilance and negative symptoms suggests that cognitive remediation therapy may have potential value in routine interventions, which may help improve participant functional outcomes. Network analysis studies also suggest that childhood trauma influences functional outcomes through different symptom pathways (22), and interventions should consider the specificity of trauma subtypes.

### Limitations

This study has several limitations. First, the sample size was relatively small, particularly in the clinical subgroup analysis where the non-trauma group had only 20 participants, resulting in insufficient statistical power and potential false-negative findings. Second, the cross-sectional design precludes causal inferences; the relationships between childhood trauma, cognition, and symptoms may be bidirectional or may share common causes. Third, childhood trauma was assessed using self-report questionnaires, which may be subject to recall bias, particularly as participants’ memory accuracy may be affected by the illness itself. Notably, cognitive impairment and negative symptoms in schizophrenia may influence the accuracy of retrospective reporting. For instance, participants with negative symptoms or depressive states might perceive or report their childhood experiences differently, potentially introducing bias. Fourth, information on antipsychotic medication dosage and duration of untreated psychosis (DUP) was not collected; medication may influence cognitive function, and this confounding factor could not be controlled. Fifth, biological markers (such as cortisol, inflammatory factors, neuroimaging data) were not included, limiting the ability to explore underlying mechanisms. Sixth, the sample size for mediation analysis was further reduced (50 cases), potentially affecting the stability of results.

### Future directions

Future research should advance in the following directions: First, larger sample sizes are needed to improve statistical power, and longitudinal designs should be employed to track the dynamic effects of trauma on cognition and symptoms. Second, multimodal data (structural/functional imaging, genetics, inflammatory markers) should be incorporated to explore the biological pathways through which childhood trauma affects cognition and symptoms. Third, potential mediators such as emotion regulation strategies, social support, and self-esteem should be examined. Fourth, nonlinear relationships and threshold effects between trauma and cognition should be explored (17) to identify precise targets for clinical intervention. Fifth, targeted cognitive remediation intervention studies should be conducted to verify the efficacy of improving attention function on negative symptoms. Sixth, interaction effects and cumulative effects of different trauma subtypes should be considered (11) to construct more comprehensive risk models. Seventh, network analysis approaches should be employed to reveal complex pathways among trauma, cognition, and symptoms (22), providing a basis for individualized intervention.

## Conclusions

This study suggests that participants with first-episode schizophrenia reported higher levels of childhood trauma and exhibited widespread cognitive impairment compared with healthy controls. Different childhood trauma subtypes were differentially associated with cognitive function and clinical symptoms, with physical neglect showing the strongest association with attention/vigilance deficits and overall cognitive performance. Attention/vigilance was significantly associated with negative symptoms but did not mediate the association between physical neglect and negative symptoms. These findings highlight the potential importance of routinely assessing childhood trauma and cognitive function in individuals with first-episode schizophrenia and suggest that further investigation of trauma-informed and cognition-focused interventions in this population may be valuable.

## Data Availability

The datasets generated and/or analyzed during the current study are not publicly available due to the following restrictions: the ethical approval obtained for this study (Approval No. (2015) No. 72-2015127FS-2) specifically prohibits the public deposition of participant data in order to protect patient confidentiality and privacy. Furthermore, the informed consent obtained from all participants and/or their legal guardians did not include provisions for sharing individual-level data in public repositories. Access to the data is therefore restricted. However, de-identified and aggregated data may be made available from the corresponding author upon reasonable request, subject to approval by the Ethics Committee of Beijing Anding Hospital, Capital Medical University. Requests to access the datasets should be directed to Xian-Bin Li, xianbinli@ccmu.edu.cn.
